# Improving the clinical assessment of consciousness with advances in electrophysiological and neuroimaging techniques

**DOI:** 10.1186/1471-2377-10-11

**Published:** 2010-01-29

**Authors:** Jodie R Gawryluk, Ryan CN D'Arcy, John F Connolly, Donald F Weaver

**Affiliations:** 1 Department of Psychology, Dalhousie University, Halifax, Canada; 2 National Research Council, Institute for Biodiagnostics (Atlantic) Halifax, Canada; 3 Department of Radiology, Dalhousie University, Halifax, Canada; 4 Department of Linguistics & Languages, McMaster University, Hamilton, Canada; 5 Queen Elizabeth II Health Sciences Centre (Neurology), Halifax, Canada; 6 Department of Medicine (Neurology), Dalhousie University, Halifax, Canada; 7 Department of Chemistry, Dalhousie University, Halifax, Canada

## Abstract

In clinical neurology, a comprehensive understanding of consciousness has been regarded as an abstract concept - best left to philosophers. However, times are changing and the need to clinically assess consciousness is increasingly becoming a real-world, practical challenge. Current methods for evaluating altered levels of consciousness are highly reliant on either behavioural measures or anatomical imaging. While these methods have some utility, estimates of misdiagnosis are worrisome (as high as 43%) - clearly this is a major clinical problem. The solution must involve objective, physiologically based measures that do not rely on behaviour. This paper reviews recent advances in physiologically based measures that enable better evaluation of consciousness states (coma, vegetative state, minimally conscious state, and locked in syndrome). Based on the evidence to-date, electroencephalographic and neuroimaging based assessments of consciousness provide valuable information for evaluation of residual function, formation of differential diagnoses, and estimation of prognosis.

## Review

### Introduction

Consciousness is a poorly-defined concept, the meaning of which is more a matter of debate than an issue of certainty [1-4]. While philosophy has focused on the mind-body problem, and psychology has focused on knowledge of experience [1], remarkably little attention has been paid to the practical problems that arise from our inability to rigorously evaluate consciousness in the clinical setting. Given the myriad of common disorders that alter consciousness, the need for more sophisticated clinical assessment methods is an important and pragmatic issue.

The dilemma of assessing consciousness is also of public interest - and the public is looking towards medical science for insights. This public fascination with consciousness was well exemplified by the media attention focused upon the medical/legal/ethical problems of the Terri Schiavo and Terry Wallis cases [5]. These cases highlighted the need to assess an individual's level of consciousness beyond simply observing their behavioural status. More recently, the dramatic increase in survivable brain injuries occurring during military conflicts is also emphasizing the need for improved tools with which to assess consciousness [6,7].

Regrettably, the time-honored structural imaging techniques (computed tomography, magnetic resonance imaging) and crude behavioural assessments (Glasgow Coma Scale) that are routinely used to assess altered consciousness are inadequate. While other more sophisticated measures exist (e.g., JFK Coma Recovery Scale - Revised), many of these tests rely on the observation of behaviours (e.g., motor and/or communicative responses) that may be impaired in brain injured people. Indeed, such behaviours are often decoupled from consciousness as a direct result of the brain injury. Accordingly, there is a critical need to improve the clinical evaluation of consciousness using non behavioral based, physiologically based measures. Although ignored for routine clinical application, electrophysiological measures such as evoked potentials and event-related potentials or ERPs (derived from electroencephalography, which measure "brain waves") address this need. In this respect, electroencephalography and related methods represent an option that provides valuable clinical insight while being more accessible than other functional imaging modalities (e.g. functional magnetic resonance imaging). This report examines the potential clinical utility of an electrophysiological approach to the assessment of consciousness. Such electrophysiological indicators of consciousness have emerged from both diagnostic and prognostic studies, which support the interplay of these two key clinical questions. Please note that this report is not intended to be an exhaustive review of the literature. Rather, we describe representative examples illustrating how the assessment of consciousness may be improved with electrophysiological as well as neuroimaging techniques.

### States of Consciousness

Consciousness is a complex brain centered state of subjective experience. Although various models exist, consciousness is commonly defined by the dual aspects of wakefulness and awareness (of both the external environment and the inner self): wakefulness refers to the sub-state that permits open eyes and a degree of motor arousal (*i.e*. wakefulness defines the level of consciousness); awareness refers to the sub-state that enables experience of thoughts, memories, and emotions (*i.e*. awareness defines the content of consciousness) [8]. Although wakefulness and awareness are intimately connected - in general, one has to be awake to be aware - it is possible to identify circumstances under which they are dissociated: in complex partial seizures wakefulness can occur without awareness; in rapid eye movement sleep it is possible to be aware but not awake.

Currently, four diagnostic levels are used to describe the spectrum of disordered consciousness: coma, vegetative state, minimally conscious state, and locked-in syndrome (it should be noted that although these terms are among the most discussed and familiar, a wide range of terms exist to describe patients across the spectra used to describe consciousness - e.g., acute confusional state, amnestic state, and obtunded). While these four terms do not completely describe all patients, they provide a useful classification starting point that is widely used in the literature.

Coma (a state of deep, unarousable unconsciousness) typically follows either brainstem injury or bilateral hemispheric damage [9]. Individuals in coma have an absence of both wakefulness and awareness.

**Figure 1:**
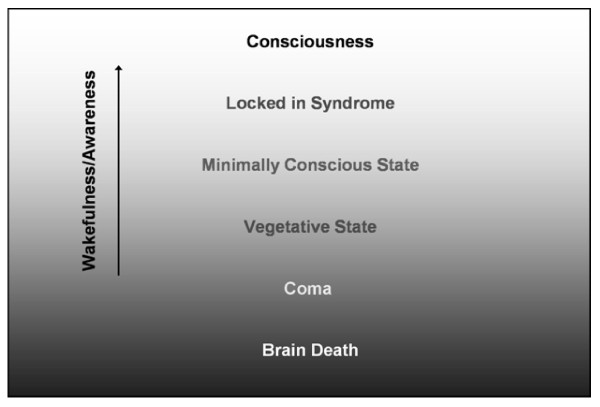
**States of Consciousness by level of wakefulness/awareness**.

From coma, a person may transition into a vegetative state (See Figure 1) [10,11]. This condition is characterized by wakefulness without awareness. Vegetative people have their eyes open and retain sleep and wake cycles, yet are unaware of themselves or their surroundings; they may even spontaneously grimace, cry or smile [8]. A vegetative state typically occurs when the brainstem is intact, but the cortex is extensively damaged [10,12], although thalamic lesions are commonly reported in this condition as well [13,14]. Not surprisingly, the vegetative state is difficult to evaluate as an altered level of consciousness.

The concept of a minimally conscious state is a relatively new diagnostic entity. Minimally conscious state is a condition in which consciousness is severely altered but some behavioural evidence of awareness remains; i.e., wakefulness with significantly diminished awareness [15]. Evidence of awareness may be demonstrated through proper use of common objects (e.g. a comb) or through non-verbal communication. The minimally conscious state can persist indefinitely or progress to full consciousness [15,16].

Another level of consciousness is locked-in syndrome, a condition associated with injury to the ventral pons [17,18]. Locked-in syndrome is characterized by intact wakefulness and awareness, but with quadriplegia and expressive anarthria; thus the afflicted individual has preserved consciousness, but, because of an inability to produce behavioural responses, appears to be unconscious [17]. This state typically follows an acute stage of impaired consciousness. Locked-in syndrome is unique in that patients in this disordered consciousness state have been able to relay their experiences. One prominent case of locked-in syndrome is that of Bauby, who provided a personal account of his experiences; Bauby describes witnessing discussions about his condition, while being helplessly unable to tell anyone that he was awake, aware and "really in there" [19]. His writings eloquently highlight a critical problem - the lack of effective clinical assessments for awareness/consciousness.

The seriousness of the consciousness assessment problem is demonstrated by the situation in which almost half of cases are misdiagnosed. Specifically, examination of the failings of conventional diagnostic approaches has yielded a misdiagnosis rate as high as 43% [20-22]. The Glasgow Coma Scale remains the "gold standard" for routine assessment of consciousness, but relies exclusively on behavioural responses [23,24]. Likewise the Disability Rating Scale also involves basic behavioural functions like eye opening, communication and motor response [25]. Although other measures of consciousness exist, all are based on behavioural signs of consciousness that are subjectively observed. The search for objective physiological measures of consciousness yielded electrophysiological approaches, which were soon followed by functional imaging approaches [26]. While both approaches can provide valuable information, the current paper focuses on electrophysiology as this technology is readily available across a range of clinical settings and thus provides a practical tool for the consciousness assessment problem [27].

### Advances in Functional Neuroimaging

Functional imaging (as opposed to structural imaging, which produces "brain pictures") describes the broad array of techniques used to assess brain function from a physiological perspective (as opposed to anatomical) [28]. One of the most well known approaches is functional magnetic resonance imaging [29,30]. Using functional magnetic resonance imaging, it is possible to track fluctuations in blood oxygenation in order to localize functionally active regions in the brain. When assessing consciousness, for example, functional magnetic resonance imaging has been used to examine activation differences between blocks of normal speech and reversed speech, making it possible to isolate regions involved in higher-level auditory processing [31,32].

Though less well known, electrophysiology represents a more established clinical option, in which evoked potentials and event-related potentials are derived from scalp-recorded electroencephalography. Evoked potentials and event-related potentials are responses that occur during the presentation of or in response to stimuli and provide an on-line record of information processing in the brain [33]. The term evoked potentials (EPs) generally refers to sensory processing responses, whereas, event-related potentials (ERPs) refers to perceptual and cognitive processing responses. In terms of nomenclature, both tend to be named according to their polarity and latency; thus, an N100 component is a negative-going waveform that peaks around 100 ms after the stimulus.

Sensory EPs are most commonly used for clinical assessment of basic sensory functions. Brainstem auditory evoked potentials (BAEPs) occur in the 10 ms range and are often employed in the assessment of coma. The absence of an intact brain stem response is indicative of a poor prognosis for recovery [34]. Sensory EPs also include somatosensory evoked potentials (SEPS), middle-latency auditory evoked potentials (MLAEPs), and visual EPs (VEPs) that occur in the 30 ms range and are used to evaluate primary sensory cortices. Cognitive ERPs are used to evaluate higher level functions like attention, memory, and language, which make them well-suited to assessing aspects of consciousness [35]. One of the most well known ERPs is the P300, which is elicited to an improbable or "oddball" stimulus that is embedded within a train of standard stimuli. (*eg*, auditory tones) [36-38].

ERP studies focusing on the assessment of conscious awareness have frequently examined four specific components - the N100, the mismatch negativity (MMN), the P300, and the N400 [35-39]. The N100 indexes sensory/perceptual functions during visual, auditory and somatosensory processing [40,41]. The MMN, a negative-going waveform occurring around 150-250 ms, has been linked to perceptual processing of deviant auditory stimuli that occurs below the level of conscious awareness [42]. The P300, which also occurs to deviant or odball stimuli, is thought to reflect higher level processing, such as immediate memory [37]. It is often known as the "Ah Hah!" response. Another important cognitive ERP component is the N400 [43,44]. The N400 is commonly observed following sentences that end with semantically inappropriate words ("He takes coffee with cream and socks"). The semantic violations can be used to assess language comprehension, by examining whether an N400 is present when comparing sentences with and without appropriate endings [45-49].

### Physiologically Based Evaluations of Consciousness

Electrophysiological approaches can be used to assess consciousness (*i.e*. awareness) across the spectrum of pathologies associated with disordered consciousness.

#### Coma

BAEPs are often employed in the assessment of coma, with the absence of response being indicative of a poor prognosis [50]. Additionally, the absence of cortical components of SEPs and of MLAEPs can also be strong indicators of a poor prognosis [51,52]. Although not currently implemented for routine use, cognitive ERPs have shown promise in determining prognosis. Fischer et al. evaluated 350 comatose patients with auditory EPs and ERPs (N100 and MMN components). They found that the MMN response was the strongest predictor of functional recovery [50]. Strikingly, 88.6% of individuals with the MMN response progressed towards awakening, corresponding with other studies, which show that the MMN can predict progression towards improved levels of consciousness [34,53,54]. Furthermore, recent meta-analysis indicated that the MMN and P300 are significantly more accurate than the N100 at predicting awakening [55]. In accordance with this, another study by Fischer et al., demonstrated that a novelty P300 elicited by the subjects own name can be used to enhance the sensitivity of assessment with the MMN and thereby increase prognostic utility in comatose patients [56].

#### Vegetative State

**Figure 2:**
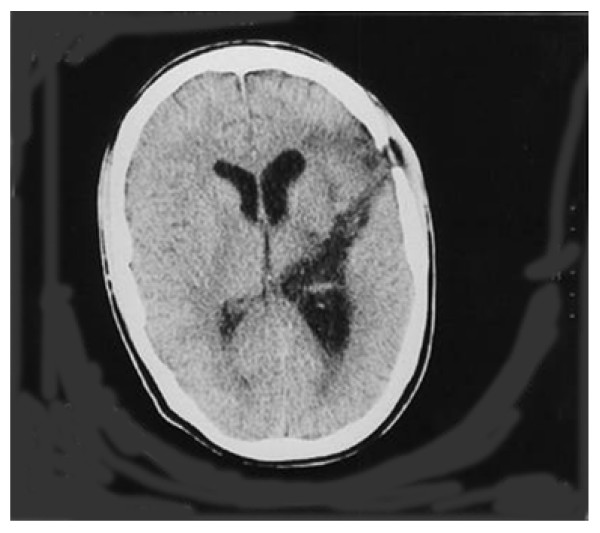
**Axial CT scan of a 21 year old male who was stabbed in the left temporal lobe. Scan was taken 4 weeks post-injury and showed no difference from original scans (adapted from Connolly et al., 1999)**.

**Figure 3:**
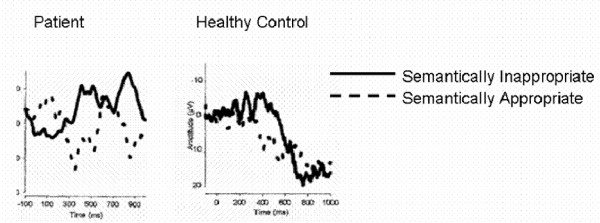
**ERP recordings from a 21 year old patient diagnosed with vegetative state and a matched healthy control. Both the patient and control show an N400 response to semantically inappropriate sentences that were spoken (adapted from Connolly et al., 1999)**.

Because vegetative state precludes verbal and motor responses, patients must be evaluated with alternative means, such as ERPs. Connolly *et al*. used cognitive ERPs in the case of man with a left temporal lobe knife wound whose ability to comprehend language was unknown (Figure 2) [47]. The task involved visual and auditory presentation of semantically appropriate and inappropriate sentences. The data from the auditory task showed a clear N400 response, indicating that awareness was intact and illustrating that cognitive ERPs can be used to assess awareness in individuals unable to communicate (Figure 3) [47].

Wijnen et al. used the MMN response to predict functional recovery in vegetative state. Ten patients were assessed with the MMN and the P300 [57]. The results indicated that larger MMN components were associated with improved signs of consciousness. Importantly, Wijnen et al. found that individuals with higher amplitudes and shorter latencies in their MMN measurement were more likely to have a higher level of consciousness two years later. Electrophysiological evaluations can be augmented by functional imaging techniques to further enhance the assessment of consciousness. For instance, functional magnetic resonance imaging and positron emission tomography studies of patients in altered levels of consciousness typically assess passive activation patterns, such as patient responses to their own name [32]. Recently, Owen et al. pursued a unique approach when evaluating a 23 year old female in a vegetative state [58]. After confirming that the patient was able to hear auditory stimuli, she was given two mental imagery tasks to assess level of awareness. She was asked to imagine playing a game of tennis and to imagine visiting all of the rooms in her home (spatial navigation imagery). Both tasks elicited activation patterns that were indistinguishable from those produced by controls (supplementary motor areas during tennis task and parahippocampal gyrus, posterior parietal cortex and lateral premotor cortex during home task). Therefore, the authors concluded that the patient retained the ability to respond to language despite being diagnosed with vegetative state (although the possibility remains that this patient may have been minimally conscious and misdiagnosed as vegetative, especially given the high misdiagnosis rate) [58]. Studies like this highlight the ability to evaluate intent, which is a critical component of awareness and represents a different approach than measures that evaluate indicators for information processing (e.g., ERPs) [12].

#### Minimally Conscious State

Most of the work on the minimally conscious state has used functional imaging to differentiate it from vegetative state. Boly et al. utilized ^15^O-radiolabeled water positron emission tomography to examine differences between minimally conscious state (N = 5) and vegetative state (N = 15). The patients were tested with a series of auditory 'clicks' approximately one month after being admitted to hospital [59]. The authors found widespread activation in the bilateral auditory cortex in minimally conscious state patients compared to vegetative state patients. In addition, minimally conscious state patients demonstrated stronger functional connectivity between secondary auditory cortex and posterior temporal and prefrontal association areas [59]. Di et al. used functional magnetic resonance imaging to study the differential diagnosis between vegetative state (N = 7) and minimally conscious state (N = 4) by presenting the patients own name (spoken by a family member). All of the minimally conscious state patients showed activation in primary and higher order auditory cortex. Interestingly, all but two vegetative state patients showed less activation than minimally conscious state patients, with these two patients subsequently progressing to minimally conscious state within three months. These results provide further support that functional neuroimaging of auditory processing may be used to distinguish different levels of consciousness [60].

#### Locked-in Syndrome

Family members, nurses and therapists are often more likely than physicians to realize that people with locked-in syndrome are conscious [61]. Electrophysiological approaches, such as cognitive ERPs, can diagnose this state at an early time point. Onofrj et al. studied four individuals suspected of having locked-in syndrome using the P300 [62]. The results indicated that a prototypical P300 waveform could be reliably detected in each of these people. The finding of residual cognitive function supported the diagnosis of locked-in syndrome as opposed to vegetative state [62]. The Onofrj et al. study is consistent with the results of other work using cognitive ERPs to evaluate awareness in locked-in syndrome [63].

### Limitations

Electrophysiological assessments of consciousness are quite promising, yet a number of limitations to clinical implementation have been identified. Given that these tools still reside within the research realm, there is limited normative data. In particular, more research needs to be done on the diagnostic/prognostic accuracy of specific components within a test battery [63,64]. In general, electrophysiological and neuroimaging techniques are prone to type II errors rather than type I errors, meaning that such assessments may underestimate cognitive functioning. A potential explanation put forth by Neumann and Kotchoubey is fluctuations in arousal/alertness [65]; given this, a practical solution may be to assess patients on more than one occasion. There are also technical challenges related to recording reliable, artifact free EEG/ERPs in clinical settings (e.g., noisy environments and ease of implementation). However, advances in both hardware and software are actively being designed and implemented to address these issues. New amplifiers for portable applications exist and methods have been developed for the quantification of waveform data for user-independent clinical use. With the coming advances on all of these fronts, it will be possible to begin assessing the reliability and validity of electrophysiological measures directly within the clinical environment. This represents the critical next step to improving the practical assessment of consciousness.

## Conclusions

Electrophysiological approaches, particularly when combined with functional imaging, can provide objective, clinically relevant assessments of consciousness - even in the presence of concomitant motor and communication problems. The MMN response shows promise in determining prognosis in coma and vegetative state; the MMN and the P300 have diagnostic utility across a range of different levels of awareness. The N400 is particularly useful for indexing high level awareness, such as intact comprehension. When used comprehensively in conjunction with other functional imaging modalities (functional magnetic resonance imaging, positron emission tomography), electrophysiological evaluations have demonstrated value in ascertaining differential diagnosis, residual function, and prognosis. Moreover, cognitive ERPs can be used to evaluate information processing which, in turn, can be used as an indicator of preserved function in brain-injured individuals.

## Competing interests

The authors declare that they have no competing interests.

## Authors' contributions

JRG and RCND were responsible for drafting the manuscript. JFC and DFW critically revised the draft for intellectual content. All authors provided final approval of the version to be published.

## Pre-publication history

The pre-publication history for this paper can be accessed here:

http://www.biomedcentral.com/1471-2377/10/11/prepub
